# The Effectiveness of Internet-Based Cognitive Behavioral Therapy as a Preventive Intervention in the Workplace to Improve Work Engagement and Psychological Outcomes: Protocol for a Systematic Review and Meta-analysis

**DOI:** 10.2196/38597

**Published:** 2023-01-19

**Authors:** Wasana Luangphituck, Plernpit Boonyamalik, Piyanee Klainin-Yobas

**Affiliations:** 1 Boromarajonani College of Nursing Suphanburi Praboromarajchanok Institute Suphanburi Thailand; 2 Department of Public Health Nursing Faculty of Public Health Mahidol University Bangkok Thailand; 3 The Alice Lee Centre for Nursing Studies Yong Loo Lin School of Medicine National University of Singapore Singapore Singapore

**Keywords:** systematic review, internet-based, cognitive behavioral therapy, work engagement, psychological outcomes, employees, workplace, web-based, occupational health, mental health, stress, depression, anxiety, eHealth, digital health

## Abstract

**Background:**

Mental health has become an increasingly significant issue in the workplace. Non–health care workers are experiencing increased levels of psychological symptoms in their workplaces, especially during the COVID-19 pandemic, which limited social interactions and health service access. These conditions have a negative effect on employees’ mental health and may also be associated with work-related outcomes, such as reduced levels of work engagement. Cognitive behavioral therapy (CBT) is one of the most effective methods used for treating workers with mental illness and preventing work-related psychological outcomes. The delivery of internet-based CBT (iCBT) has been established as a result of both technological improvements that have influenced health promotion and prevention components, and limited social contact and health service access.

**Objective:**

The purpose of this systematic review is to synthesize the best available evidence concerning the preventive effect of iCBT on employees.

**Methods:**

A systematic search will be conducted across 12 electronic databases, including a hand search for main journals and reference lists. Randomized controlled trials testing the effects of iCBT on psychological outcomes and work engagement among employees will be eligible. Initial keywords will cover the concepts of employees, workers, non–health care personnel, internet-based, web-based, eHealth cognitive behavioral interventions, stress, depression, anxiety, and work engagement, and then a full search strategy will be developed. Following titles, abstracts and the full text will be screened for assessment against the inclusion criteria for the review. Search results will be fully reported and presented per Preferred Reporting Items for Systematic Reviews and Meta-Analyses guidelines. Two independent reviewers will screen and extract data, appraise methodological quality using the Cochrane risk-of-bias assessment tool, and assess overall quality of evidence with the Grading of Recommendations Assessment, Development, and Evaluation approach. A random effects meta-analysis and standardized mean differences using review manager software will be applied to synthesize the effect of iCBT based on similar outcomes.

**Results:**

This protocol was registered in the International Prospective Register of Systematic Reviews in March 2022 and is now an ongoing process. The data will be analyzed in August 2022, and the review process should be completed by December 2022. All included studies will be synthesized and presented to demonstrate the effectiveness of iCBT in decreasing psychological distress and optimizing work engagement outcomes among employees.

**Conclusions:**

According to the findings of this study, iCBT therapies will be used to promote mental health concerns such as depressive symptoms, anxiety, psychological distress, stress, insomnia, and resilience among non–health care professionals. In addition, the results will be used to ensure the policy related to reducing psychological distress and optimizing work engagement in the workplace.

**International Registered Report Identifier (IRRID):**

PRR1-10.2196/38597

## Introduction

### Background

The extended COVID-19 pandemic has had an impact on the mental health of both health care workers and non–health care personnel [1]. Previous research has found a significant frequency of depression (34.6%), anxiety (42.3%), and psychological distress (65.1%) among workers during the COVID-19 pandemic [2]. This means that psychological distress has turned out to be more noticeable among employees in the workplace [3] during the COVID-19 pandemic. Psychological distress is the unique, uncomfortable, emotional state experienced by an individual in response to a specific stressor or demand that results in harm, either temporary or permanent, to the person [4]. Employees in various workplaces commonly face stress, anxiety, depression, attention-deficit/hyperactivity disorder, and bipolar illnesses. The symptoms indicate psychological distress involving insomnia, fatigue, difficulty concentrating, etc [5]. Long working periods, inappropriate salaries, job stress, an unhealthy working environment, and several risk factors in the workplace could also have a serious impact on one’s mental health [6].

Previous systematic reviews have found that psychological distress such as stress, depression, and anxiety have been endured by a large number of employees around the world [3,5]. Those symptoms could prompt weakened prosperity such as sleep problems, fatigue, burnout, and manifest diseases including the consequence of work engagement outcomes. This not only impacts individuals’ psychological well-being and reduces their capacity [6-8] but also leads to a financial burden on companies and governments [3,9]. Work engagement is also an important point among employees because it is linked to job performance and productivity. Work engagement is defined as a good, satisfying state of mind related to work that is characterized by vitality, dedication, and immersion [10]. Work engagement has been related to significant individual and organizational outcomes [11].

To minimize these consequences, a greater understanding of interventions that facilitate the improvement of psychological problems among employees is essential for protecting and promoting the mental health of workers during the prolonged COVID-19 pandemic [1,8,12].

### Cognitive Behavioral Therapy Intervention

Cognitive behavioral therapy (CBT) is one of the most effective interventions to not only treat mental illnesses [13] but also prevent work-related psychological outcomes [14,15]. Internet-based CBT (iCBT) is a form of psychotherapy based on CBT principles, which is delivered through the internet by an individual or software that is not physically present with the client [13]. The delivery of iCBT was developed because of today’s advanced technology, which has impacted everyday life, including aspects of health promotion and prevention, as well as providing an advantage during the COVID-19 crisis, which limited social interaction and health service access. Internet-delivered programs are named differently as *online interventions*, *web-based interventions*, *e-therapy*, *eHealth*, and *computerized cognitive behavioral therapy* [16]. In recent years, iCBT interventions have yielded techniques, which have been developed and tested in different forms for mental problems including depression and anxiety [13,17-19].

### Rationale for Performing a Systematic Review

Previous systematic reviews have suggested that group iCBT interventions enhanced work performance among mental health workers [20]; reduced stress, anxiety, and depression among health personnel [21]; and reduced subsequent sickness-related absenteeism from work among adults with common mental disorders [14]. In addition, several researchers have examined the effectiveness of iCBT in improving outcomes for people with chronic illnesses and chronic mental disorders [18,19,22]. However, all of these existing reviews focused on health personnel and persons with mental health disorders and thus results may not be generalized to healthy workers and non–health care workers. Furthermore, most reviews used anxiety, depression, and insomnia as psychological outcomes but overlooked other work-related variables such as work engagement. Consequently, a comprehensive review and meta-analysis investigating the effectiveness of iCBT interventions on these outcomes among healthy workers is necessary.

A preliminary search of the Cochrane Database of Systematic Reviews, The JBI Database of Systematic Reviews and Implementation Reports, and the previous registration of an ongoing study in International Prospective Register of Systematic Reviews (PROSPERO) found no recent systematic reviews on this topic in progress. Therefore, the aim of this systematic review and meta-analysis is to evaluate the efficacy of iCBT as a preventive intervention on work engagement and psychological outcomes for nonclinical workers.

### Review Objective and Question

An objective of this systematic review is to synthesize the best available evidence concerning the preventive effect of iCBT on employees. Review questions are as follows: (1) in comparison to controls, what is the preventive effect of iCBT in improving psychological outcomes (including stress, anxiety, depression, insomnia, and resilience) among healthy non–health care employees? (2) In comparison to controls, do iCBT interventions, as preventive interventions, enhance work engagement among healthy non–health care employees?

## Methods

The proposed systematic review will be conducted in accordance with the Cochrane Handbook for Systematic Reviews of Interventions [23].

### Protocol Registration

A summary of the protocol and supplemental data were registered in PROSPERO (CRD42022320589) [24] to provide a methodological outline of this systematic review and meta-analysis.

### Eligibility Criteria

#### Study Selection

This systematic review will admit studies that (1) involved participants who were healthy non–health care workers of any sociocultural background, such as office workers, military personnel, and teachers; (2) examined programs using iCBT or iCBT with co-interventions; and (3) included our primary outcomes, including psychological distress, depression, stress, anxiety, insomnia, and resilience, and examined our secondary outcome—work engagement. Due to logistic issues, we considered studies reported in English without any year restriction.

Exclusion criteria for this systematic review are studies that (1) involved participants who were health care professionals and those with mental disorders or physical illnesses who were different in context from non–health care workers, and (2) tested only face-to-face CBT.

#### Intervention

For this review, the iCBT program, as a preventive intervention, is defined as a psychotherapy program based on CBT principles such as cognitive restructuring, behavioral activation, and behavioral experiments, delivered via the internet, remotely, or through mobile-based platforms in an individual or group formats. This is in accordance with the aim of this review, which is to test the effectiveness of the core method of iCBT interventions on psychological distresses and work engagement outcomes.

#### Comparator

This review will consider studies that compare intervention groups with no intervention controls, active controls, placebo controls, and usual-care groups.

#### Outcomes

The studies that evaluate the effect of iCBT on any aspect of psychological outcomes including depression, stress, anxiety, insomnia, and resilience will be considered primary outcomes. This review will also consider studies of the effect on work engagement, which is operationalized as a good, satisfying state of mind associated with work, characterized by vitality, dedication, and immersion as a secondary outcome.

### Types of Studies

This review will consider only randomized controlled trials. Studies reported in English will be included without any year restriction.

### Information Sources

The 6 electronic databases to search published studies include PubMed, CINAHL, Embase, Ovid, MEDLINE, Scopus, and IEEE Xplore. Ongoing clinical trials will be searched in the clinical trial registry. Sources of unpublished studies or gray literature to be searched include Google Scholar, ProQuest Dissertations, and Theses Global and Ethos. The date when each database will be last searched is May 2022.

### Search Strategies

The search strategy targets published and unpublished studies. The Peer Review of Electronic Search Strategies checklist [25] will be used in the search strategy. An initial limited search of PubMed will be undertaken to identify articles on the topic. Initial keywords will cover the PICO framework [26], which includes *participants* (employees, workers, and non–health care personnel), *intervention* (internet-based, web-based, and eHealth cognitive behavioral interventions), and *outcomes* (such as depression, anxiety, and work engagement; Multimedia Appendix 1). Boolean and proximity operators will be used to combine the search terms. Subsequently, the text words contained in the titles and abstracts of relevant articles, and the index terms used to describe the articles were used to develop a full search strategy for CINAHL, Embase, Ovid, MEDLINE, and Scopus. The search strategy, including all identified keywords and index terms, will be adapted for each included database and information source. A librarian will be consulted to ensure the comprehensiveness of the search strategy. The reference list of all included sources of evidence will be screened for additional studies. A hand search for the main journal such as the *Journal of Occupational Health*, the* *Journal *of *Occupational Health Psychology, and the Journal of Medical Internet Research will be also conducted.

### Study Selection

Keywords and Medical Subject Headings terms (ie, *Internet-based cognitive behavioral therapy program* AND *Employees*) were piloted on PubMed. Following the search, all identified citations will be collated and uploaded into EndNote version 9 [27,28], and duplicates will be removed. Titles and abstracts will then be screened by 2 independent reviewers for assessment against the inclusion criteria for the review. The full text of selected articles will be assessed in detail against the inclusion criteria by 2 independent reviewers. Reasons for exclusion of sources of evidence in the full text that do not meet the inclusion criteria will be recorded. Any disagreements that arise between the reviewers at each stage of the selection process will be resolved through discussion or with an additional reviewer. The results of the search will be reported in full in the final systematic review and presented in a Preferred Reporting Items for Systematic Reviews and Meta-Analyses flow diagram [29].

### Assessment of Methodological Quality

A pilot test with 6 studies will be undertaken to guarantee consistency between the 2 reviewers responsible for the selection and data extraction process. Then, 2 independent reviewers will assess each eligible study for methodological quality prior to inclusion in the review, using the Cochrane risk-of-bias tool for randomized trials [30]. Potential biases in studies may include a selection bias, performance bias, detection bias, attrition bias, and selective report bias. Each domain will be rated as “Yes,” “No,” or “Unclear.” All included studies will be processed in the next phase regardless of risk-of-bias scores. The study authors will be contacted to obtain information if needed.

The overall quality of evidence will be assessed using the Grading of Recommendations Assessment, Development and Evaluation method. Findings for each outcome will be rated as high, moderate, low, or very low quality. Items include study design, risk of bias, imprecision, inconsistency, indirectness, and publication bias. Any disagreements that arise between the reviewers will be resolved through discussion or with other reviewers.

### Data Extraction

Data will be extracted from studies included in the review by 2 independent reviewers. A pilot test will be conducted on 6 studies to ascertain agreement between the reviewers. The data extracted will include specific details about the populations, interventions, comparison, outcomes, findings, and measurement of significance to the review objective. Any disagreements that arise between the reviewers will be resolved through discussion or with a third reviewer. Authors of papers will be contacted to request missing or additional data, where required.

### Data Synthesis and Presentation

Outcomes will be analyzed as continuous and dichotomous outcomes whenever possible. Meta-analyses will be performed using the random effects model for outcomes using Review Manager (RevMan) [31] computer software. For continuous outcomes, the standardized mean difference and 95% CIs for the means will be calculated. A *P* value of less than .05 will indicate statistical significance for the overall effect. Heterogeneity of effect sizes across studies will be determined by significant chi-square values and an *I*^2^ value of >60%. Subgroup analyses will be performed [32]. A Funnel plot will be used to examine publication bias if there are 10 or more studies included in the meta-analysis. An asymmetry funnel plot will substantiate the presence of publication bias. A meta-analysis will be conducted with studies that report on the same outcomes. 

## Results

This study was supported by the Department of Public Health Nursing, Faculty of Public Health, Mahidol University, Thailand, in January 2022. This systematic review protocol was started in February 2022 and registered in PROSPERO in March 2022. As of August 2022, the data were analyzed, and the final report of the review were completed at the end of December 2022.

The characteristics of the included studies and the description of iCBT interventions will be summarized. The findings from the meta-analysis will show and describe the ability of iCBT to change psychological distress and work engagement outcomes among employees. When quantitative synthesis is not possible, a narrative synthesis of the results will be used.

## Discussion

Here we provide a short summary of the main findings related to the aim and hypothesis of this review. The discussion will be motivated by the fact that the iCBT intervention will benefit selected outcomes based on the existing pool effect size of all included studies and how they add new findings to these fields. In addition, here we shall explain why the effective iCBT interventions yielded the results that they did and how they would be supported by the current evidence and comparison to prior work.

There will be strengths and limitations to this review. In contrast to previous reviews, we will concentrate on healthy non–health care workers. The new evidence of preventive psychological and work engagement outcomes is to be included in our review. Only randomized controlled trials were included in the study because they are regarded as the most suitable research designs.

The meta-analysis will be carried out in order to pool the effect size among individual studies for each outcome. This result could predict the evidence supporting the use of iCBT interventions in clinical practice. The limitations to this review will be explained in terms of the possibilities for future research, such as the generalizability of the findings, confirming further results, and clarifying more outcomes in future research.

The findings from this review will provide evidence for clinical and policy implications that internet delivery has the potential to improve the benefits of CBT in terms of reducing psychological consequences and increasing work engagement level. This conclusion will be used to assist in making decisions regarding implementing iCBT techniques as a preventive intervention for healthy non–health care employees in clinical practice, such as the variety of program characteristics, including content and duration, which will be examined for use in enhancing employee outcomes. The efficacy of iCBT will also be used to improve the negative impacts of psychological conditions and support high levels of work engagement in the workplace.

According to the findings of this study, iCBT therapies will become a strategy for promoting mental health issues among healthy non–health care workers, such as depressive symptoms, anxiety, psychological distress, stress, insomnia, and resilience. The summary of this review will also support future decision-making regarding the adoption of an internet-based intervention to reduce psychological discomfort among employees. Importantly, the findings will be used to ensure workplace policies that aim to alleviate the negative impacts of psychological conditions.

## Appendix

Multimedia Appendix 1 Summary of systematic search strategies.

## Abbreviations

CBT: cognitive behavioral therapy
iCBT: internet-based cognitive behavioral therapy
PROSPERO: International Prospective Register of Systematic Reviews
