# Supplementary material for: The Effectiveness of Internet-Based Cognitive Behavioral Therapy as a Preventive Intervention in the Workplace to Improve Work Engagement and Psychological Outcomes: Protocol for a Systematic Review and Meta-analysis
Source: JMIR Res Protoc. 2023 Jan 19;12:e38597. doi: 10.2196/38597 (PMC9896353; doi:10.2196/38597)
Supplement: Multimedia Appendix 1 [file resprot_v12i1e38597_app1.pdf]

Multimedia Appendix 1

Summary of systematic search strategies

A preliminary search of PubMed Search

Date: March 2022

PCC	Search terms	Record identified
Participants	1. Workers	885,160
	2. Employees	817,989
	3. Non-healthcare workers	221
	4. Occupational groups	711,379
	5. Workplace employees	25,667
	6. 1 or 2 or 3 or 4 or 5	981,782
Interventions	7. Cognitive behavioral therapy	52,039
	8. Internet-based intervention	8,415
	9. Computer-based intervention	9,127
	10. Internet-based cognitive behavioral therapy	1,198
	11. Web-based cognitive behavioral therapy	670
	12. E-health cognitive behavioral therapy	122
	13. 7 or 8 or 9 or 10 or 11 or 12	67,949
Outcomes	14. Stress	1,143,812
	15. Stress disorders	138,783
	16. Anxiety	286,235
	17. Anxiety disorders	159,048
	18. Depression	570,733
	19. Depression disorders	317,670
	20. Insomnia	30,653
	21. Sleep initiation	29,095
	22. Resilience	52,329
	23. Psychological Resilience	14,643
	24. Work engagement	14,260
	25. Workplace engagement	14,759
	26. Employees engagement	28, 297
	27. Worker participation	15,593
	28. 14 or 15 or 16 or 17 or 18 or 19 or 20 or 21 or 22 or 23 or 24 or 25 or 26 or 27	1,882,471
	29. 6 and 13 and 28	1,874,621

Initial keywords will cover PICO framework: participants (Employees, Workers, Non-healthcare personnel), intervention (Internet-based, web-based, and e-health cognitive-behavioral interventions), and outcomes (such as stress, depression, anxiety, insomnia. resilience and work engagement).

Limit search: 1) English 2) Title Abstract and keywords
